# Nonlinear Complexity Analysis of Brain fMRI Signals in Schizophrenia

**DOI:** 10.1371/journal.pone.0095146

**Published:** 2014-05-13

**Authors:** Moses O. Sokunbi, Victoria B. Gradin, Gordon D. Waiter, George G. Cameron, Trevor S. Ahearn, Alison D. Murray, Douglas J. Steele, Roger T. Staff

**Affiliations:** 1 Aberdeen Biomedical Imaging Centre, University of Aberdeen, Aberdeen, United Kingdom; 2 Department of Nuclear Medicine, Aberdeen Royal Infirmary, Aberdeen, United Kingdom; 3 Medical Research Institute, University of Dundee, Dundee, United Kingdom; 4 Centre for Basic Research in Psychology, Universidad de la Republica, Montevideo, Uruguay; 5 Cardiff University Brain Research Imaging Centre, Cardiff University, Cardiff, United Kingdom; 6 Institute of Psychological Medicine and Clinical Neurosciences, Medical Research Council Centre for Neuropsychiatric Genetics and Genomics, Cardiff School of Medicine, Cardiff University, Cardiff, United Kingdom; University Of Cambridge, United Kingdom

## Abstract

We investigated the differences in brain fMRI signal complexity in patients with schizophrenia while performing the Cyberball social exclusion task, using measures of Sample entropy and Hurst exponent (H). 13 patients meeting diagnostic and Statistical Manual of Mental Disorders, 4th Edition (DSM IV) criteria for schizophrenia and 16 healthy controls underwent fMRI scanning at 1.5 T. The fMRI data of both groups of participants were pre-processed, the entropy characterized and the Hurst exponent extracted. Whole brain entropy and H maps of the groups were generated and analysed. The results after adjusting for age and sex differences together show that patients with schizophrenia exhibited higher complexity than healthy controls, at mean whole brain and regional levels. Also, both Sample entropy and Hurst exponent agree that patients with schizophrenia have more complex fMRI signals than healthy controls. These results suggest that schizophrenia is associated with more complex signal patterns when compared to healthy controls, supporting the increase in complexity hypothesis, where system complexity increases with age or disease, and also consistent with the notion that schizophrenia is characterised by a dysregulation of the nonlinear dynamics of underlying neuronal systems.

## Introduction

Schizophrenia can be characterized as deficits in the interaction between thought, emotion and behaviour resulting from inappropriate selection, ordering and sequencing of behavioural elements [1]. Problems of social interaction are a central feature of schizophrenia [2]. Positive symptoms of schizophrenia including hallucinations and delusions have social components while negative symptoms are exhibited as loss of motivation, social withdrawal and self-neglect [3]. The social impairments in schizophrenia have been linked to poor clinical outcomes [4] but there is limited understanding of the underlying mechanisms underpinning these impairments. Recently, a few studies have investigated the neural mechanisms underlying social impairment in schizophrenia using fMRI [2].

**Figure 1:**
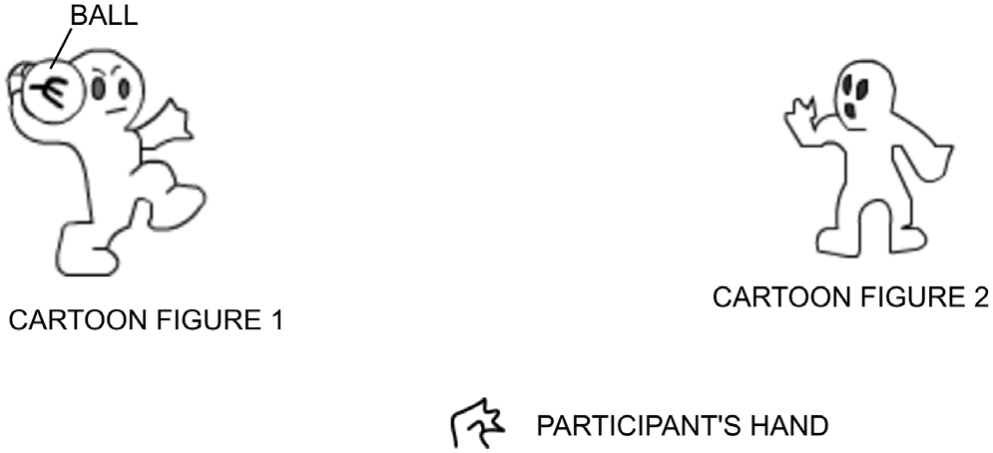
The Cyberball social exclusion task.

The fMRI paradigm that has most frequently been used to study social exclusion is the “Cyberball” task [5]. In this task, participants play a ball-passing game with two cartoon figures and the participant is included and excluded from the game at different times (Figure 1). Some brain regions have been found to show responses to social exclusion. They are the ventral anterior cingulate cortex (vACC) and the medial prefrontal cortex (mPFC) [6]. Also, the ventrolateral PFC has been found to show responses to social exclusion [7], [8]. The discrimination of these brain regions suggests that they are relevant for social information processing.

Information processing in biological systems such as the human brain operates at multiple levels. At the cellular level, it occurs as a result of the dynamic communicatory activities of the nervous system which can be influenced by physical, chemical and electrical stimuli [9]. It also operates at the network level, especially in fMRI signals where high resolution voxels contain many thousands of neuronal connections. Activities such as information processing in biological systems are governed by thresholds and saturation phenomena [9]. When these thresholds are exceeded, saturation sets in giving rise to nonlinear effects. Biological systems typically exhibit complex behaviour with nonlinear dynamic properties [10]. Nonlinearity, a necessary condition for chaotic behaviour is present in many dynamic systems existing in nature, such as the human brain [9]. One important manifestation of nonlinear effects at the network level is what can be thought of in terms of hemodynamic “refractoriness” [11], in which a prior stimulus modulates the response to a subsequent stimulus that is proximate in time. This means that responses at high-stimulus presentation rates saturate and, in some instances, show an inverted U behaviour. This modulation represents an interaction, over time, between the response to successive stimuli and results in reduced responsiveness at high-stimulus frequencies. This behaviour appears to be specific to BOLD effects [11]. Recent developments in the study of nonlinear dynamics have provided novel methods that may facilitate a better understanding of complex systems in biology and medicine.

Investigators have argued that the pathway of change in the behaviour and physiology of an organism with age and disease can either result in a decrease or an increase in the complexity of the system’s output. Complexity can be defined as the difficulties arising when describing or predicting a signal [12]. Normal physiology requires an intricate network to control function effectively. These networks incorporate a mix of integrations, differentiation, feedback loops, and other regulatory mechanisms that enable an organism to perform multiple and varied activities. Lipsitz and Goldberger [13], [14] argued that with ageing and disease, there is a loss of complexity in the dynamics of many integrated physiological processes of an organism. Vaillancourt and Newell postulate that the directional change in output complexity of a physiological or behavioural system with ageing or disease depends on the system having an underlying fixed point or an oscillatory attractor determining output. An attractor is the state to which a system returns to after perturbation [15]. The fixed-point attractor system generates more complexity in the output when it is healthy and optimal performance is maintained. When the system is failing, output complexity is reduced and optimal performance may not be maintained. An example of a fixed point attractor system is blood pressure regulation which occurs about a fixed-point intrinsic dynamic (homeostatic process). In those systems where the attractor is oscillatory the opposite is true. The system output increases in complexity when it is failing. An example of an oscillatory attractor is the circadian rhythm.

The historical development of the concepts of complexity has centred on measuring regularity using various metrics based on nonlinear time series analysis techniques. One of these techniques is the correlation dimension, which has been shown to be lower in Alzheimer’s disease (AD) patients than control subjects [16]–[18]. Also, the first Lyapunov exponent has been used to characterize nonlinear dynamics [19] where AD patients have significantly lower first Lyapunov exponent values than controls [18], [20]. The shortcomings of these techniques are that they require a large data set [21] and assume that the time series is stationary [22], which is normally not true for biological data.

An alternative solution is to compute the entropy of the time series. Entropy measures the randomness and predictability of a stochastic process and in general increases with greater randomness. Sample entropy (SampEn) computes the negative logarithm of the conditional probability that two similar sequences remain similar at the next point, where self matches are not included in calculating the probability [23]. A lower value of SampEn indicates lower complexity of the time series, while a higher value indicates higher complexity. Another alternative approach is to compute the fractal complexity of the time series. The Hurst exponent, H, is a measure of the fractal complexity (predictability) or persistence of fractal processes such as fractional Gaussian noise or Brownian motion [24]. The values of the Hurst exponent, H range between 0 and 1. A time series can be classified into three categories based on the value of H; (1) H = 0.5 indicates a random white noise series; (2) 0<H<0.5 indicates a rough anticorrelated series and; (3) 0.5<H<1 indicates a positively correlated series. These estimates of complexity can be used to probe different aspects of complex signals which may be affected by ageing and disease, and can be applied to a range of physiological measures.

The application of nonlinear dynamic analysis techniques in schizophrenia using fMRI has been very limited to date [25], [26] and to the best of our knowledge these techniques have not been used to investigate social exclusion in schizophrenia. The present study investigated the changes in complexity of brain fMRI signals of patients with schizophrenia while performing a social exclusion task (a modified version of the Cyberball paradigm that aimed to minimise the confounding effect of expectation violation) [2], using measures of Sample entropy and Hurst exponent. Brain signals from patients with schizophrenia have been hypothesized to constitute a complex dysregulation of neurobiological and behavioural patterns rather than a simple up and down regulation [27]. Therefore, we hypothesise that patients with schizophrenia would exhibit higher complexity due to dysregulation in neural responses when compared to healthy controls.

## Materials and Methods

### Participants

The Grampian Local Research Ethics Committee (now North of Scotland Research Ethics Committee), National Health Service (NHS) Grampian, Aberdeen approved the present study. The ethics approved information sheet was given to all the patients and healthy controls. It was suggested that participants discuss the study with other people and take several days to decide on any questions. The participants were then invited to a screening interview where one of the researchers (DJS) had a discussion with them to determine if they understood the study. At the interview sessions, they were encouraged to ask any questions they might have, which would then be answered. Only the participants that understood the task, wished to participate in the study and met the eligibility criteria were recruited to the study. All participants provided written informed consent by signing the consent form themselves. 13 patients (11 male) meeting Diagnostic and Statistical Manual of Mental Disorders, 4th Edition (DSM IV) criteria for schizophrenia (mean age; 41.23±11.78) and 16 healthy controls (7 male) (mean age; 41.56±11.92) were recruited. Exclusion criteria were any other neurological disorder (e.g. epilepsy, gross structural brain abnormality), claustrophobia, or other DSM IV Axis I or II diagnosis.

The patients antipsychotic medications at the time of scanning were the following (dose/day): clozapine 250–900 mg, quetiapine 250–700 mg, olanzapine 20 mg, risperidone 6 mg and chlorpromazine 500 mg, depot pipothiazine palmitate 50 mg, four weekly and depot flupenthixol decanoate 200 mg, three weekly. Two patients were also receiving long term antidepressant medication because of previous episodes of depressive illness: sertraline 50 mg and citalopram 20 mg.

### Schizophrenia Syndrome Severity Scores

Immediately before the fMRI acquisition, all the patients were assessed using the Positive and Negative Syndrome Scale (PANSS) [28], which is used as an index of the schizophrenia syndrome severity. The mean PANSS scores are shown in Table 1.

**Table 1:** Participants’ characteristics, SampEn, Hurst exponent, PANSS, CPZE and Social distress measures.

	Control group	Patients with schizophrenia	Significance
Age (years)^a^	41.56±11.920	41.22±11.780	p = 0.941
Sex (M/F)	7/9	11/2	
SampEn^b^	1.456±0.030	1.598±0.033	p = 0.004
SampEn after adjusting for only age differences^b^	1.456±0.031	1.598±0.034	p = 0.004
SampEn after adjusting for only sex differences^b^	1.476±0.029	1.574±0.033	p = 0.043
SampEn after adjusting for both age and sex differences together^b^	1.476±0.030	1.573±0.033	p = 0.048
Hurst exponent^b^	0.779±0.013	0.687±0.015	p = 0.000
Hurst exponent after adjusting for only age differences^b^	0.779±0.013	0.688±0.014	p = 0.000
Hurst exponent after adjusting for only sex differences^b^	0.768±0.012	0.701±0.013	p = 0.002
Hurst exponent after adjusting for both age and sex differences together^b^	0.767±0.011	0.702±0.013	p = 0.001
PANSS_positive^a^		13.230±2.390	
PANSS_negative^a^		12.310±5.880	
PANSS_general^a^		22.230±6.860	
PANSS_total^a^		47.770±13.120	
CPZE^a^		491.000±254.700	
Social distress (Averaged score)^a^	3.775±1.245	3.776±2.597	p = 0.999
Belonging^a^	6.569±1.449	4.731±3.901	p = 0.092
Self-esteem^a^	4.959±1.775	5.000±3.200	p = 0.965
Meaningful existence^a^	1.171±1.961	1.748±2.958	p = 0.540
Control^a^	2.401±2.483	3.175±3.606	p = 0.501

a Values are – Mean ± Standard Deviation;

b Values are – Mean ± Standard Error; SampEn - Sample entropy; PANSS – Positive and Negative Syndrome Scale; CPZE - chlorpromazine equivalents.

### Antipsychotic Medication Equivalents

The different antipsychotic medication doses were placed on a common dimension using the chlorpromazine equivalents (CPZE), which is a numeric scale. Table 1 shows the mean chlorpromazine equivalents.

### Social Distress Behavioural Measures

Both patients and controls were assessed using a self-report ‘social distress’ rating questionnaire [5] after fMRI acquisition. The average social distress score was estimated from four primary social ‘needs’: belonging, self-esteem, meaningful existence and control [5]. A 0 to 10 point question was used to assess each social need, ranging from 0 (not at all) to 10 (very much). The ‘social distress’ assessment followed those closely described by Gradin et al [2]. The mean scores of the ‘social distress’ ratings are shown in Table 1.

### Brain Imaging Procedures

Functional MR images were acquired with a T_2_* weighted gradient echo echo-planar imaging sequence (EPI) in the axial plane using a GE Medical Systems Signa 1.5 T MRI scanner. A total of 30 axially orientated 5 mm thick contiguous sequential slices were obtained for each of 244 volumes using a TR of 2.5 s, TE of 30 ms, flip angle of 90°, field of view of 240 mm and matrix 64×64. A total of 240 volumes of fMRI data remained after discarding the first four volumes to allow for signal conditioning. To help maintain the subject’s attention, fMRI was done using a standard head coil while performing the “Cyberball” social exclusion task.

### The “Cyberball” Social Exclusion Task

The “Cyberball” social exclusion task is shown in Figure 1. All participants performed a version of the “Cyberball” task during fMRI scanning. This task has already been described in detail by Williams et al. [5] and Eisenberger et al. [7]. In Figure 1, the participants represented by an animated hand, play a ball passing game with two cartoon figures. The participants pressed one of two buttons to pass the ball to each of the cartoon figures in turn. Each of the cartoon figures also pass the ball to either the participant or the other cartoon figure in turn. Following the approach taken by Gradin et al. [2], the ratio at which the participant was excluded in the task was varied from 0% exclusion to 100% exclusion. The “Cyberball” task was explicitly divided into 17 blocks with the following percentage levels of exclusion: 0, 25, 50, 75, 100, 75, 50, 25, 0, 25, 50, 75, 100, 75, 50, 25 and 0 [2]. The time that the cartoon figures took to pass the ball was varied randomly to give the impression that they represented ‘real’ people making decisions. The task was executed in 10 minutes.

### Image Pre-processing

The fMRI image pre-processing was performed using SPM8 software (The Wellcome Department of Imaging Neuroscience, UCL, London, UK). The fMRI data were realigned to correct for head movement distortion. Temporal high pass filtering was performed (128 seconds) to reduce low frequency noise. Also, spatial smoothing was performed to suppress noise and effects due to residual differences in functional and gyral anatomy during inter-subject averaging using the full-width at half maximum (FWHM) of the Gaussian smoothing kernel [8 8 8]. Each voxel time series was standardized to a mean of zero and standard deviation of unity.

### Calculation of SampEn

SampEn is a modification of the approximate entropy (ApEn) algorithm [23]. It was proposed in order to reduce the bias of ApEn, where self-matches were excluded from the ApEn algorithm, (*i ≠ j*) and (*1≤ i ≤ N-m*) i.e. vectors are not compared to themselves. The ApEn algorithm counts each sequence as matching itself to avoid the occurrence of ln(0). SampEn was developed and characterized as a new family of measures. It has the advantage of being less dependent on time series length, and is able to demonstrate relative consistency over a broader range of possible r, m, and N values under circumstances where ApEn does not [23]. SampEn has been used to characterize the nonlinear features of heart rate (HR) time series for three recumbent positions [29]. It has also been used to study abnormal HR characteristics of reduced variability early in the course of neonatal sepsis, where it has been shown that SampEn of the neonatal HR falls before the clinical diagnosis of sepsis [30], indicating its value as an “early warning” of clinical deterioration. It was applied to MEG signals from Alzheimer’s disease patients [31]. Also, it has been applied to MEG signals from ADHD patients [32] and a recent study of resting state fMRI signals from adult ADHD patients [33]. SampEn can be applied to discriminate both stochastic processes and noisy deterministic systems. The estimation of SampEn for a time series of length N 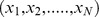 is given as [33]:
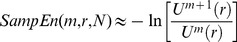


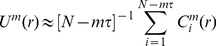
(1)Where



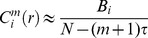


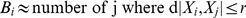
(2)

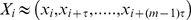
(3)

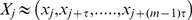
(4)

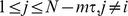

*N* is the number of time points, *m* specifies the pattern length, r defines the tolerance value and 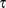 is the time delay as shown in Eq. (1). In Eq. (2), the two patterns *i* and *j* of m measurements of the time series are similar if the difference, d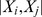 between any pair of corresponding measurements of 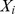 and 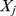 is less than or equal to r. In Eq. (3) and (4), 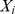 and 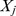 are pattern vectors (length m) whose components are time delayed versions of the elements in the original time series with time delay, 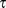.

Whole brain SampEn maps for each participant of both groups were generated on a voxel by voxel basis using the same approach as Sokunbi et al [34] on a MATLAB and C platform. SampEn was estimated with the following parameters; N = 240 of fMRI time series, m = 2, 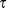 = 1 and r = 0.32 (see Appendix S1) multiplied by the SD of the fMRI time series. SampEn was computed for the whole brain at a threshold of 0.1 times the maximum signal to exclude voxels being calculated outside the brain. The mean whole brain SampEn value for each participant was calculated.

### Estimation of Hurst Exponent

Hurst exponent provides a measure of long-range correlations of a time series. Several methods for estimating the Hurst exponents are available, including Hurst rescaled range analysis, which is the oldest and most common [35], autocorrelation analysis [36], Fourier analysis [36], [37] and maximum likelihood estimators [38]. Each of the above methods suffers from biases and slow convergence, a large dataset is required to reduce the bias. However, two methods have performed consistently better, requiring smaller datasets and producing less bias [39]. These are dispersional analysis and Detrended Fluctuation analysis.

Dispersion analysis entails the measurement of the variance or standard deviation of a signal at a succession of different levels of resolution [39]. The different levels are obtained by grouping data points and replacing each with the group average, taking successively larger groups, which is equivalent to reducing the resolution. Dispersional analysis can be regarded as a strong method for characterizing biological or natural time series, which generally show long-range positive correlation [39]. Biological signals may be combinations of fractal and periodic components [40]. Not many such signals have been examined for their fractal nature. Dispersional analysis has been fairly applied to examine the regional flow distribution in the heart [41], the lung [42] and the kidney [43]. In the present study, we apply it to fMRI data of patients with schizophrenia during social exclusion.

The dispersional relationship of a given signal of length N 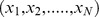 is given as:
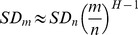


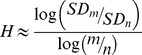
(5)In Eq. (5), *H* denotes the Hurst exponent, *SD* is the variance or the standard deviation, *m* specifies the element size used to calculate SD, and n is the arbitrarily chosen reference size, which is the length over which an average is obtained. Here, N = 240 volumes of fMRI time series, m = 2 and n = 1. A linear regression on the log-log plot was performed and the calculated slope is the best estimate of H. Whole brain H maps were produced from these estimates of H. The estimates of H were obtained using MATLAB.

### Statistical Analysis

Conventional statistical analysis was performed on the mean whole brain SampEn and H values of each subject in both groups using the Statistical Package for Social Sciences (SPSS 18.0; Chicago, IL, USA) software. Independent t-tests and General Linear Model analyses with correction for only age, only sex and both age and sex differences were performed.

The SampEn and H maps of each subject were normalised to a standard echo planar imaging (EPI) template, and regional analyses performed using the two- sample t-test in SPM8, comparing the control and schizophrenic groups after correcting for both age and sex differences together at a family-wise error (FWE) corrected cluster level significance [44] of p<0.05 and threshold p = 0.005.

We performed the receiver operating characteristic (ROC) analyses [45] for the mean whole brain SampEn and H values in SPSS.

Correlations between the PANSS (positive, negative, general and total) scores and the estimates of signal complexity (SampEn and Hurst exponent) at whole brain and regional levels were tested. Also, correlations between the CPZE equivalents and the estimates of signal complexity (SampEn and Hurst exponent) at whole brain and regional levels were tested. Furthermore, correlations between the average social distress scores and the estimates of signal complexity (SampEn and Hurst exponent) at whole brain and regional levels were tested. These whole brain correlations were performed using Pearson correlation analyses, while a multiple regression approach in SPM8 was used for the regional correlations, after correcting for both age and sex differences together.

## Results

**Figure 2:**
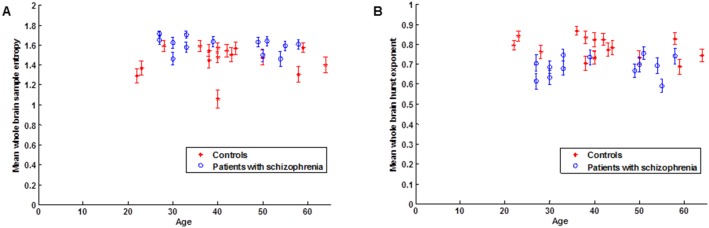
Plots of mean whole brain complexity of the individual participants with increasing age. (A) Mean whole brain Sample entropy. (B) Mean whole brain Hurst exponent. Error bars denote the standard error of the mean.

**Figure 3:**
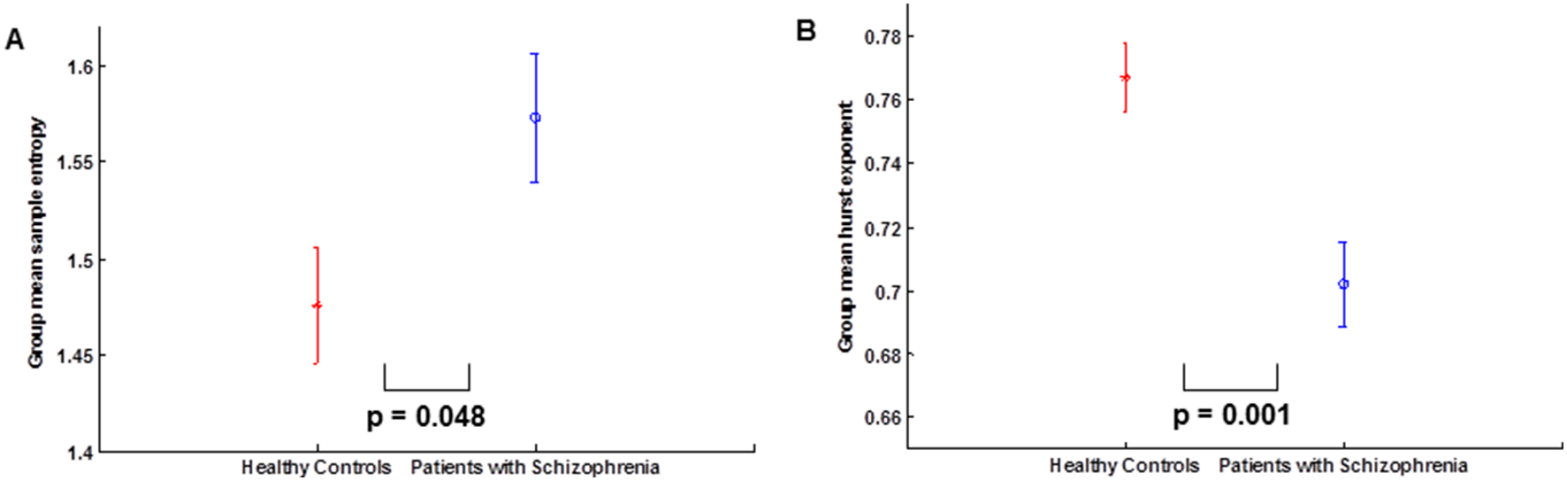
Group mean complexity differences after correcting for age and sex differences together in the GLM. (A) Group mean Sample entropy. (B) Group mean Hurst exponent.

Table 1 show the participants characteristics, SampEn, Hurst exponent, PANSS, CPZE and self-report behavioural (social distress) measures. Figure 2 (A) and (B) depicts the distribution of the mean whole brain sample entropy and mean whole brain Hurst exponent respectively, of individual participants with increasing age. The control group’s age ranges from 22 to 64 years and patients with schizophrenia from 27 to 58 years. Table 1 shows that when the General Linear Model analyses in SPSS were repeated with correction for only age differences (*p* = 0.004 and *p* = 0.000), only sex differences (*p* = 0.043 and *p* = 0.002) and both age and sex differences together (*p* = 0.048 and *p* = 0.001), the mean whole brain SampEn and mean whole brain Hurst exponent of both groups respectively, remained significantly different. Figure 3 (A) and (B) show the mean whole brain SampEn and mean whole Hurst exponent difference between the two groups respectively.

There were no significant differences in individual need scores (belonging, self-esteem, meaningful existence and control) or in the overall average social distress score between the control and patient groups (see Table 1). This shows that patients and controls were engaged with the Cyberball task and perceived the varying inclusion and exclusion effect in a similar manner.

**Figure 4:**
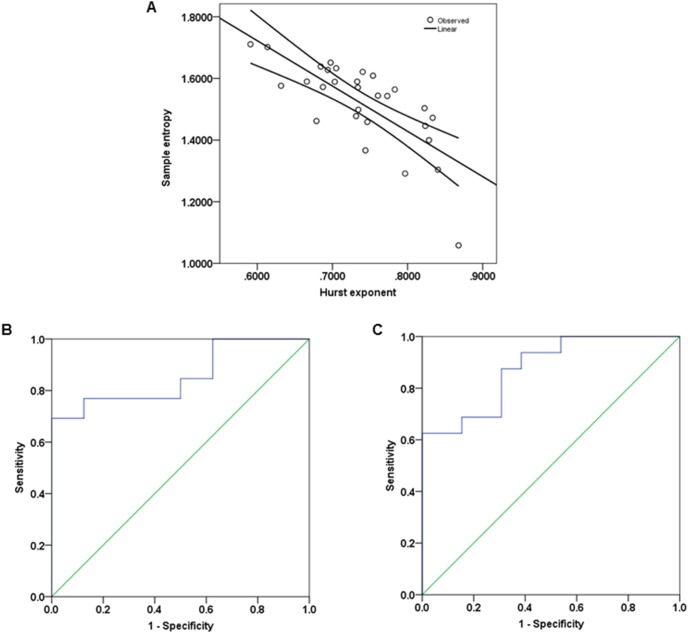
Correlation between sample entropy and Hurst exponent, and ROC curves. (A) Linear regression curve estimation between the mean whole brain sample entropy and mean whole brain Hurst exponent for the whole population. (B) ROC curve for Sample entropy. (C) ROC curve for Hurst exponent.

Figure 4(A) shows the linear regression curve estimation between the mean whole brain sample entropy and mean whole brain Hurst exponent for the whole population. The curve shows that both estimates of complexity are significantly negatively correlated (p = 0.001, r = −0.606) after correcting for both age and sex differences together. Also, we evaluated the ability of SampEn and Hurst exponent to discriminate patients with schizophrenia from controls at mean whole brain using the ROC analysis (Zweig and Campbell 1993). For Sample entropy we obtained the value for the area under the ROC curve as 0.856, sensitivity as 76.90%, specificity as 87.50% and accuracy as 82.20% at a threshold of 1.5742. The Hurst exponent produced an ROC area of 0.875, sensitivity of 68.80%, specificity of 84.60% and accuracy of 76.70% at a threshold of 0.7421. A guide to classifying the precision of a diagnostic test is related to the area under the ROC curve. For values of the area between 0.90 and 1, the precision of the diagnostic test is considered to be excellent, good for values between 0.80 and 0.89, fair for area between 0.70 and 0.79, poor when the area is between 0.60 and 0.69 and bad for values between 0.50 and 0.59. Hence, the results obtained can be considered good for both Sample entropy and Hurst exponent. Figure 4(B) and (C) show the ROC curves for the mean whole brain sample entropy and Hurst exponent respectively.

**Figure 5:**
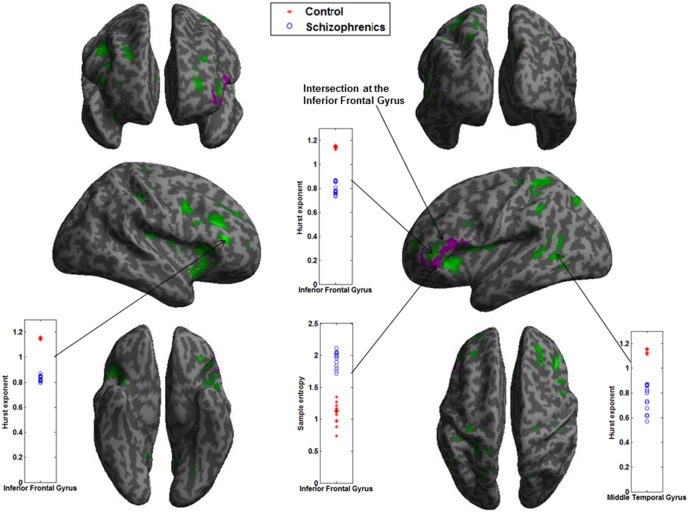
Scatter plots and rendered images showing differences between control and patients with schizophrenia after correcting for age and sex differences together. Scatter plots depict the mean SampEn and H values at different brain ROI. Rendered images show the difference in SampEn and H between the control and patients with schizophrenia. Regions shown have higher complexity in patients with schizophrenia. See table 2 for a complete list of these regions (threshold p = 0.005 and corrected cluster p<0.05).

When the data were tested regionally with a family-wise error (FWE) corrected cluster level significance of p<0.05, the results of the two-sample t-tests (p = 0.005) after correcting for both age and sex differences together are shown in figure 5. The discriminated regions in magenta and green in the images show significant differences in SampEn and Hurst exponent between the two groups. Notably, patients with schizophrenia have higher SampEn values (higher complexity) and lower H values (higher fractal complexity) compared to controls. The scatter plots of the brain areas show complete separation between the groups, as shown in figure 5. Furthermore, the group differences of both estimates of complexity intersect at the left Inferior Frontal Gyrus. Table 2 shows the anatomical location of the SampEn and Hurst exponent differences between both groups, after adjusting for age and sex differences together.

**Table 2:** Anatomical location of the regions discriminated in SampEn and Hurst exponent differences between Controls and patients with schizophrenia after adjusting for both age and sex differences together.

Estimate of signalcomplexity	Brain region	Talairachcoordinate (XYZ)	Brain label	Tissue type	Cluster p value (FWE corrected)	Voxel t value
Sample entropy (SampEn)	Frontal Lobe	−56 24 10	Left Inferior Frontal Gyrus	White Matter	0.041	4.17
		−52 38 0	Left Brodmann Area 47	Gray Matter	0.041	4.02
Hurst exponent (H)	Frontal Lobe	38 30 16	Right Inferior Frontal Gyrus	White Matter	0.000	6.24
		−24 –42 36	Left Sub-Gyral	White Matter	0.000	4.97
		36 –26 36	Right Sub-Gyral	White Matter	0.000	4.95
		−36 32 –4	Left Inferior Frontal Gyrus	White Matter	0.000	5.44
	Sub-lobar	−28 26 4	Left Extra-Nuclear	White Matter	0.000	4.52
		−30 0 20	Left Extra-Nuclear	White Matter	0.000	3.85
	Temporal Lobe	−24 –52 12	Left Sub-Gyral	White Matter	0.002	4.50
		−34 –58 0	Left Sub-Gyral	White Matter	0.002	4.09
		−46 –44 4	Left Middle Temporal Gyrus	White Matter	0.002	3.96

The location coordinates are those of the peak significance in each region (threshold p = 0.005, FWE corrected cluster p<0.05).

There were no significant (p>0.05) correlations between each PANSS (positive, negative, general and total) scores and each estimate of signal complexity (SampEn and Hurst exponent) at whole brain and regional analyses levels respectively. Also, no significant (p>0.05) correlations were found between antipsychotic dose (calculated as chlorpromazine equivalent) and each estimate of signal complexity (SampEn and Hurst exponent) at whole brain and regional levels respectively. Furthermore, there were no significant (p>0.05) correlations between the average social distress scores and the estimates of signal complexity (SampEn and Hurst exponent) at whole brain and regional analyses levels respectively.

## Discussion

This study reports global and regional differences in SampEn and Hurst exponent between patients with schizophrenia and controls while performing a social exclusion task. After adjusting for age and sex differences together, the results show that there were differences in SampEn and Hurst exponent values at whole brain and regional levels. In the SampEn analysis, patients with schizophrenia exhibited significantly higher SampEn values (higher complexity) than controls while in the Hurst exponent analysis patients exhibited significantly lower H values (higher fractal complexity) than controls. These significant differences indicate that patients with schizophrenia exhibited more complex fMRI signal than the healthy controls. Also, the significant negative correlation (p = 0.001, r = −0.606) between the two estimates shows that as the SampEn values increases (higher complexity), the H values decreases (higher fractal complexity). This confirms that both SampEn and H agree that patients with schizophrenia have more complex fMRI signal than healthy controls and that the difference between the control and the patient population is actually due to real differences and not the measure employed.

From our results, both SampEn and Hurst exponent results upheld our hypothesis of a higher complexity and are consistent with the hypothesis that brain signals from schizophrenia constitute a complex dysregulation of neurobiological and behavioural patterns rather than a simple up and down regulation [27]. These findings seem to support the increase in complexity hypothesis of Vaillancourt and Newell [15] which states that there is an increase in the output complexity of a physiological or behavioural system with ageing or disease when the system is governed by an oscillatory attractor. Increases in complexity have been previously shown to be associated with schizophrenia. Takahashi et al [46] measured the dynamic EEG signal complexity in schizophrenia subjects using multiscale entropy and found that they showed significantly higher complexity at higher time scales than in healthy controls in fronto-centro-temporal, but not in parieto-occipital regions. Post-treatment, this higher complexity decreased to healthy control subject levels selectively in fronto-central regions, while the increased complexity in temporal sites remained higher. In a study of bimanual finger movements of schizophrenia patients, it was observed that rhythmic bimanual coordination is more complex in schizophrenic patients [47]. These findings suggest that patients with schizophrenia consistently demonstrate complex behavioural and physiological output, consistent with our fMRI complexity results. If these system theory postulates are true, this would imply that there is an underlying oscillatory mechanism determining, at least in part, brain function and that this mechanism is compromised in schizophrenia.

The abnormally higher complexity may be brought about by a putative abnormal neurochemical mechanism in schizophrenia. It has been suggested that a disturbance in dopamine signalling to the prefrontal cortex may underlie abnormalities observed in this region in social cognition studies of schizophrenia [48]. Dopamine is a catecholamine neurotransmitter produced in several areas of the brain, including the substantia nigra and the ventral tegmental area of the midbrain. The higher signal complexity observed in this study may be a consequence of a failure in the feedback mechanism of the dopamine system responsible for keeping the system(s) stable.

Our analyses identified abnormalities in the frontal region of the brain previously identified in a variety of studies using different techniques. In a volumetric study in first-episode schizophrenia using tensor-based morphometry, Whitford et al [49] found that 41 first-episode schizophrenia patients showed reduced white matter volume in a number of regions at baseline relative to healthy controls. This study concluded that “Given the role that white matter plays in neural communication, the authors suggest that these white matter abnormalities may be a cause of the dysfunctional neural connectivity that has been proposed to underlie the symptoms of schizophrenia”. It may well be that the increased complexity observed in this study is a result of such dysfunctional neural connectivity. Using resting-state fMRI to measure functional connectivity and functional network topology in schizophrenia [25] the strength of functional connectivity was found to be significantly decreased, whereas the diversity of functional connections was increased. They concluded that people with schizophrenia tend to have a less strongly integrated, more diverse profile of brain functional connectivity, associated with a less hub-dominated configuration of complex brain functional networks. Frontal brain connectivity has been shown to be abnormal in patients with schizophrenia [50]. Also, Gradin et al. [2] found abnormalities in the mPFC of patients with schizophrenia.

Examining the locations in figure 5 and table 2, the results showed that both SampEn (magenta) and Hurst exponent (green) discriminated the left inferior frontal gyrus and also intersected at this location. In the frontal lobe, SampEn also discriminated the left BA 47 while Hurst exponent detected the right inferior frontal gyrus. The inferior frontal gyrus consists of Brodmann areas 44, 45 and 47, which also make up the ventrolateral Prefrontal Cortex (vPFC). The ventrolateral PFC has been reported in fMRI studies to be active in responses to social exclusion [7], [8]. We suggest that the discrimination of these regions in the frontal lobe may be a response to the Cyberball social exclusion task performed by the participants. The Hurst exponent analysis also discriminated other brain regions not detected by SampEn: the left extra-nuclear (sub-lobar region) and left middle temporal gyrus were detected in addition.

Noise is a signal of irregular frequency; it oscillates at irregular intervals with time and is the signal with the most complex dynamics and highest measured entropy [12]. It has been hypothesized that increased noise corruption in the dopamine system in schizophrenia could interfere with normal phasic responses to events [51]–[53]. The findings of higher complexity in patients with schizophrenia in this study support this hypothesis.

In the ROC analyses, the area under the ROC curve for Sample entropy (0.856) and Hurst exponent (0.875) shows that the results obtained can be considered good for both analyses. Although, Hurst exponent had a higher ROC area and discriminated more regions in the regional analysis, Sample entropy had a higher sensitivity (SampEn = 76.90% and H = 68.80%), specificity (SampEn = 87.50% and H = 84.60%) and accuracy (SampEn = 82.20% and H = 76.70%) than Hurst exponent. This suggests that Sample entropy was a more accurate discriminator and hence a better diagnostic tool in this regard.

A weakness of our study is that patients with schizophrenia were not drug naive. All patients with schizophrenia were taking some form of anti-psychotic medication, reflecting standard psychiatric practice. We found no evidence of an association between antipsychotic equivalent medication doses and the estimates of complexity (SampEn and Hurst exponent). This would suggest that dose does not have a proportional influence on complexity but does not exclude a systemic one. Another limitation was that patients with schizophrenia had a higher male to female ratio than the healthy controls. We therefore corrected for the sex differences in the GLM analysis using sex as a covariate. The lack of significance in social distress between both groups suggests that the differences in complexity between patients and controls (as estimated by SampEn and Hurst exponent) are due to schizophrenia rather than one group paying more attention to the Cyberball task than the other. These relationships suggest that our findings are associated with schizophrenia syndrome severity, independent of medication and sex differences. Other limitations of the study were a limited sample size and possibly the limited number of time points of fMRI data. Also, it is unclear whether segmenting the white matter and cerebrospinal fluid (CSF) from our complexity analyses would influence our results. This step is a subject of future evaluation. Finally, it would be interesting to replicate the analysis in future studies with a larger sample size and balanced sex ratio.

## Conclusions

To the best of our knowledge this is the first study investigating the complexity of brain fMRI signals in patients with schizophrenia while performing a social exclusion task. The results showed that patients with schizophrenia have a higher signal complexity when compared to healthy control subjects. These results are consistent with the hypothesis that schizophrenia may be brought about by an underlying dysregulation of more complex functional networks and support the increase in complexity hypothesis (second postulate) of Vaillancourt and Newell [15] where system complexity increases with age or disease.

## Supporting Information

Figure S1 ROC area for detecting mean whole brain SampEn difference between controls and patients with schizophrenia for different tolerance values, r.
(TIF)

Appendix S1 Estimation of tolerance value, r in the calculation of SampEn.
(DOCX)
