# Correction: Nonlinear Complexity Analysis of Brain fMRI Signals in Schizophrenia

**DOI:** 10.1371/journal.pone.0105741

**Published:** 2014-08-07

**Authors:** The PLOS ONE Staff

Reference 54 is omitted from the reference list. Please view reference 54 here: Pincus SM (1991) Approximate entropy as a measure of system complexity. *Proc Natl Acad of Sci U S A* 88(6): 2297-2301.
