# Supplementary figures and images for: Nonlinear Complexity Analysis of Brain fMRI Signals in Schizophrenia
Source: PLoS One. 2014 May 13;9(5):e95146. doi: 10.1371/journal.pone.0095146 (PMC4019508; doi:10.1371/journal.pone.0095146)

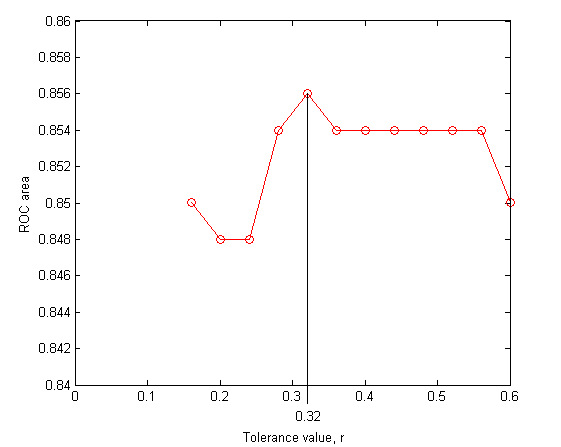

Supplement: Figure S1 — ROC area for detecting mean whole brain SampEn difference between controls and patients with schizophrenia for different tolerance values, r. (TIF) [file pone.0095146.s001.tif]
