# Supplementary material for: Nonlinear Complexity Analysis of Brain fMRI Signals in Schizophrenia
Source: PLoS One. 2014 May 13;9(5):e95146. doi: 10.1371/journal.pone.0095146 (PMC4019508; doi:10.1371/journal.pone.0095146)
Supplement: Appendix S1 — Estimation of tolerance value, r in the calculation of SampEn. (DOCX) [file pone.0095146.s002.docx]

**Appendix S1**

**Estimation of tolerance value, r in the calculation of SampEn**

An appropriate tolerance value, r is necessary for the computation of SampEn, r is essentially a filter, where the size of the filter depends on the choice of r [34]. Pincus [54] recommended the choice of r to use in the calculation of entropy as 0.1r0.2 times the standard deviation of the time series. It has been shown that this recommended range is not always appropriate for fast dynamic neural signals [12] such as fMRI [34]. In order to estimate a robust value of r for the computation of SampEn, we investigated the ability of SampEn to discriminate patients with schizophrenia from age-matched healthy controls using the receiver operating characteristic (ROC) area [45]. The ROC area is used as a guide to classify the precision of a diagnostic test. ROC areas between 0.90 and 1 means the diagnostic test is excellent, good for ROC areas between 0.80 and 0.89, fair for areas between 0.70 and 0.79, poor for ROC areas between 0.60 and 0.69 and bad for areas from 0.50 to 0.59. Using the same approach as Sokunbi et al. [33], we calculated the mean whole brain SampEn values for each of the 13 patients with schizophrenia and 16 control subjects with N=240 of fMRI time series, m= 2, $\tau$ = 1 and r ranging from 0 to 0.6 at intervals of 0.04. Only r values of 0.16 to 0.6 returned mean whole brain SampEn values, 0 to 0.12 retuned no values. The ROC areas of the mean whole brain SampEn for r ranging from 0.16 to 0.6 was calculated with SPSS software. Figure S1 shows a plot of the ROC areas for each r value. The plot shows that the ROC areas for SampEn are good for all r values, with the minimum ROC area of 0.848 occurring at r=0.20 and r=0.24 and the maximum ROC area of 0.856 occurring at r=0.32. The value of r=0.32 corresponding to the maximum ROC area was used in the main study.

**References**

54. Pincus SM (1991) Approximate entropy as a measure of system complexity. Proc

Natl Acad of Sci U S A 88(6): 2297-2301.
